# Enhanced quantum yields by sterically demanding aryl-substituted β-diketonate ancillary ligands

**DOI:** 10.3762/bjoc.14.54

**Published:** 2018-03-21

**Authors:** Rebecca Pittkowski, Thomas Strassner

**Affiliations:** 1 Physical Organic Chemistry, Technische Universität Dresden, Bergstraße, 01069 Dresden, Germany

**Keywords:** ancillary ligand, β-diketonates, photoluminescence, platinum(II) complex, quantum yield

## Abstract

Luminescent organometallic platinum(II) compounds are of interest as phosphors for organic light emitting devices. Their emissive properties can be tuned by variation of the ligands or by specific electron-withdrawing or electron-donating substituents. Different ancillary ligands can have a profound impact on the emission color and emission efficiency of these complexes. We studied the influence of sterically hindered, aryl-substituted β-diketonates on the emission properties of C^C* cyclometalated complexes, employing the unsubstituted methyl-phenyl-imidazolium ligand. The quantum yield was significantly enhanced by changing the auxiliary ligand from acetylacetonate, where the corresponding platinum(II) complex shows only a very weak emission, to mesityl (mes) or duryl (dur) substituted acetylacetonates. The new complexes show very efficient emission with quantum yields >70% in the sky-blue spectral region (480 nm) and short decay times (<3 μs).

## Introduction

Highly luminescent platinum(II) complexes have successfully been applied for lighting applications such as organic light emitting diodes (OLEDs) [1–6]. Although OLEDs are already widely used, the development of stable and efficient blue devices remains challenging [7–8]. Tetradentate [9–11], terdentate [12–15], and bidentate [16–20] cyclometalated Pt(II) complexes were recently shown to be promising phosphorescent triplet emitters in OLEDs (PhOLEDs), which emit light with high quantum yields in the blue spectral region.

The emission properties of organometallic complexes can be tuned by employing different ligand structures. For platinum(II) complexes, the influence of both cyclometalating [21–26] and auxiliary ligand [27–32] on the emission color as well as their efficiency has been demonstrated. Phenyl-substituted *N*-heterocyclic carbenes (NHCs) as C^C* cyclometalating ligands shift the emission color towards higher energy, due to the strong donor character of NHCs compared to C^N cyclometalating ligands [33–34]. Recently, it was shown that the introduction of sterically demanding aryl groups as substituents in acetylacetonate (acac) auxiliary ligands can have a positive influence on the emission properties of platinum(II) phosphors [35–38]. The use of mesityl and duryl groups enhanced the quantum yield of platinum complexes with a variety of C^C* cyclometalating ligands [18,39–41].

We herein present the synthesis and photophysical properties of two new C^C* cyclometalated platinum complexes. Both are based on the original 3-methyl-1-phenylimidazolium (MPIM) ligand system which together with the acac auxiliary ligand showed only a very low quantum yield of 7%. We introduced sterically demanding aryl substituted β-diketonate auxiliary ligands to further examine their influence on the emission properties of the resulting platinum(II) complexes.

## Results

**Scheme 1:**
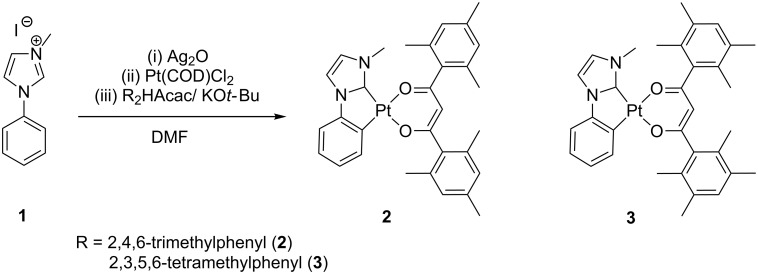
Synthesis of complexes **2** and **3**.

**Figure 1:**
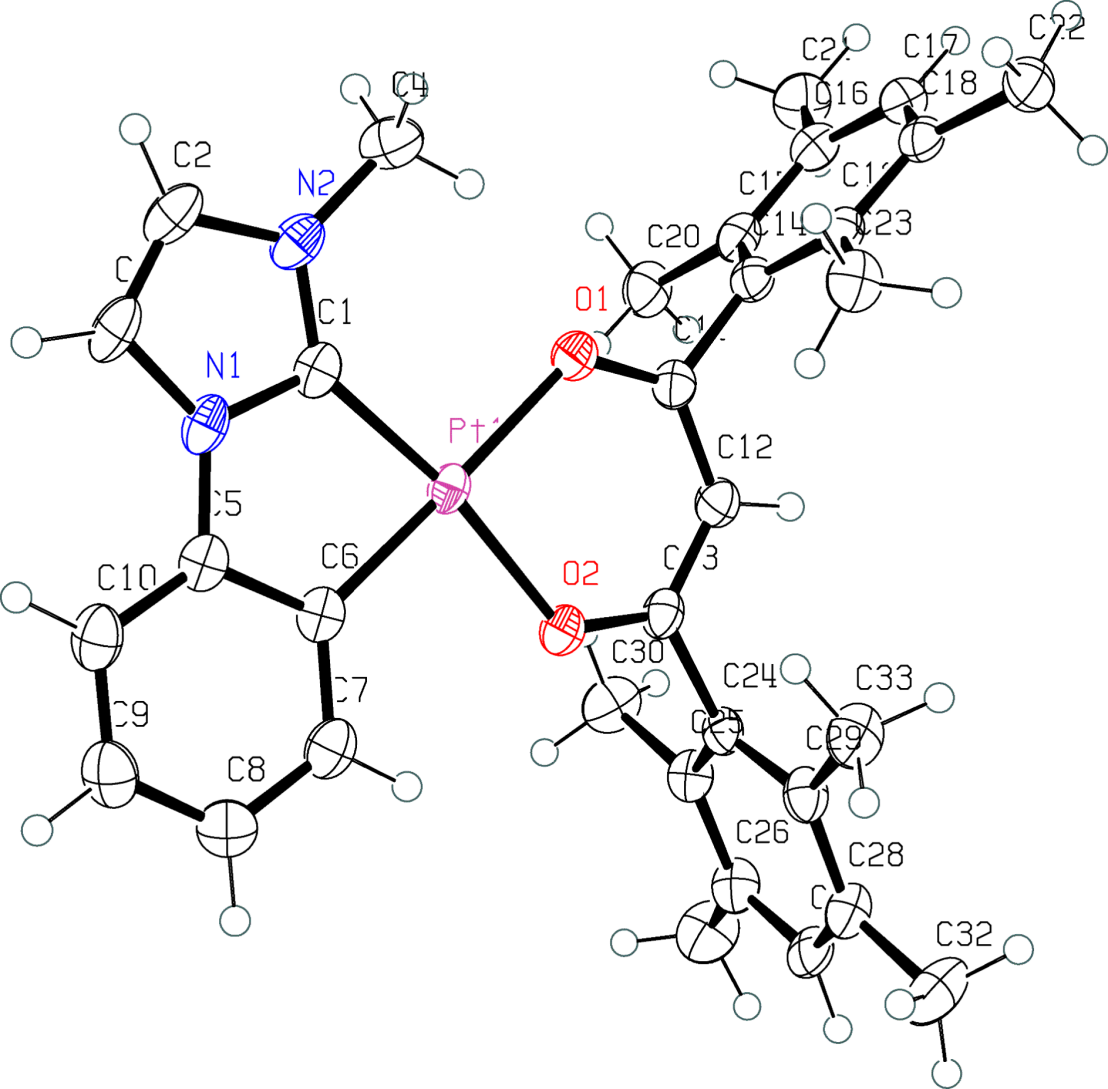
ORTEP representation of **3**. Thermal ellipsoids are drawn at the 50% probability level. Selected bond lengths (Å) and angles (deg): C(1)–Pt(1), 1.950(3); C(6)–Pt(1), 1.988(3); O(1)–Pt(1), 2.089(3); O(2)–Pt(1), 2.047(2); N(1)–C(1), 1.361(4); N(2)–C(1), 1.347(4); C(1)–Pt(1)–C(6), 80.17(13); O(1)–Pt(1)–O(2), 90.51(9); N(1)–C(1)–N(2), 104.8(3); O(1)–Pt(1)–C(1)–N(2), −2.7(4);

The mesityl- and duryl-substituted 3-methyl-1-phenylimidazole complexes **2**, Pt(MPIM)(mes) and **3**, Pt(MPIM)(dur), were synthesized from 3-methyl-1-phenylimidazolium iodide (**1**) according to a modified literature procedure (Scheme 1) [41–42]. The starting imidazolium salt **1** was prepared from phenylimidazole by addition of methyl iodide as previously described [43]. Complexes **2** and **3** were obtained as yellow solids in isolated yields of 5% and 18%, respectively (Scheme 1). They were characterized by standard methods, NMR techniques (^1^H, ^13^C, and ^195^Pt) as well as mass spectrometry (ESIMS). The purity of all compounds was verified by elemental analyses. Additionally we could unequivocally determine the structural parameters of **3** by a solid-state structure (Figure 1). Details of the structure determination are given in Supporting Information File 1, Table S1.

**Figure 2:**
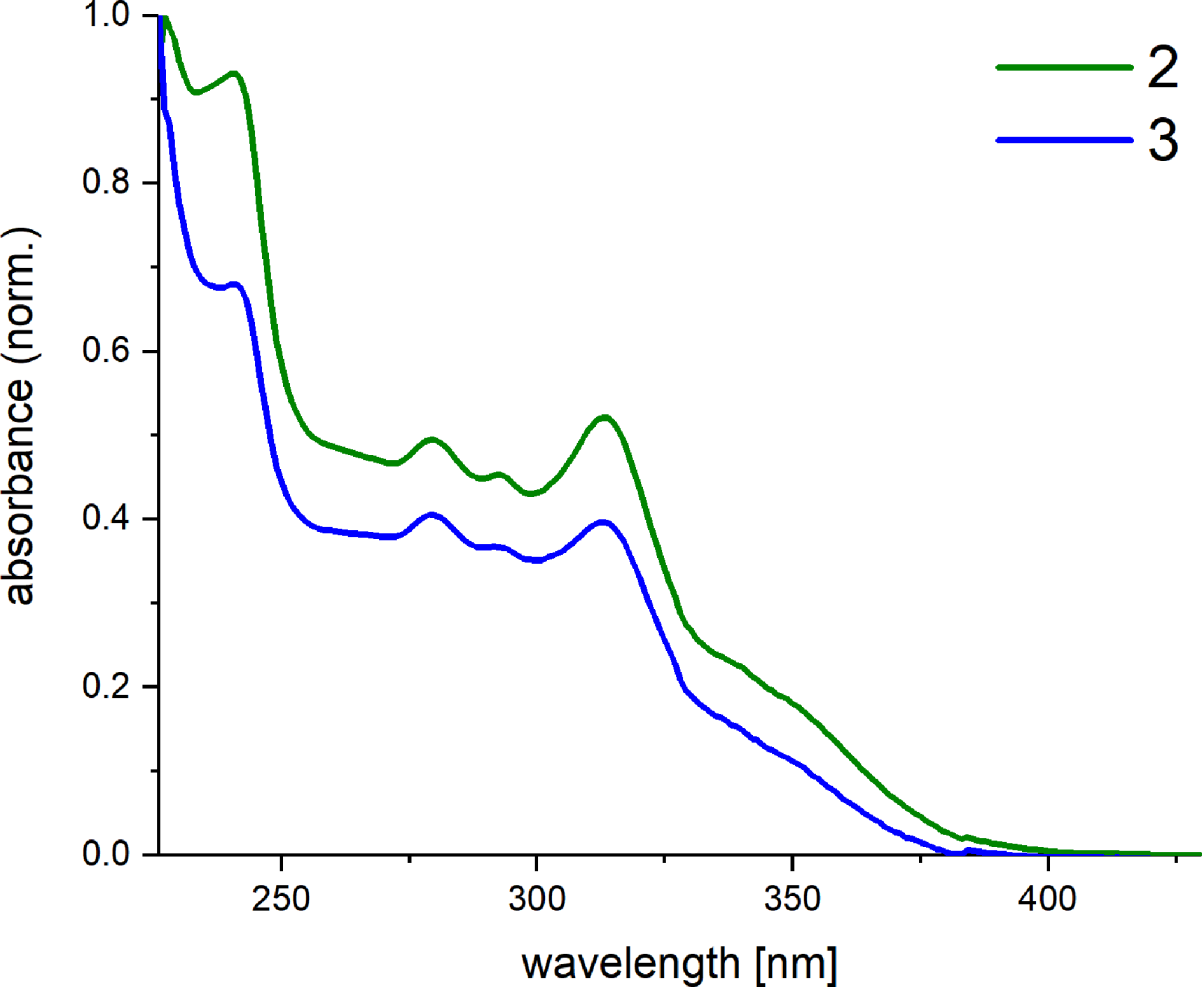
UV–vis absorption spectra of complexes **2** and **3** measured in dichloromethane at room temperature.

The absorption spectra (Figure 2) were measured in dichloromethane solution at ambient temperature. The complexes show almost identical absorption behavior with only minor deviations in the absorption intensity. Both complexes exhibit a strong absorption in the ultraviolet spectral region with an intense shoulder at 241 nm. Two weak and one more intense absorption bands are additionally located at 280 nm, 293 m, and 313 nm, respectively.

**Figure 3:**
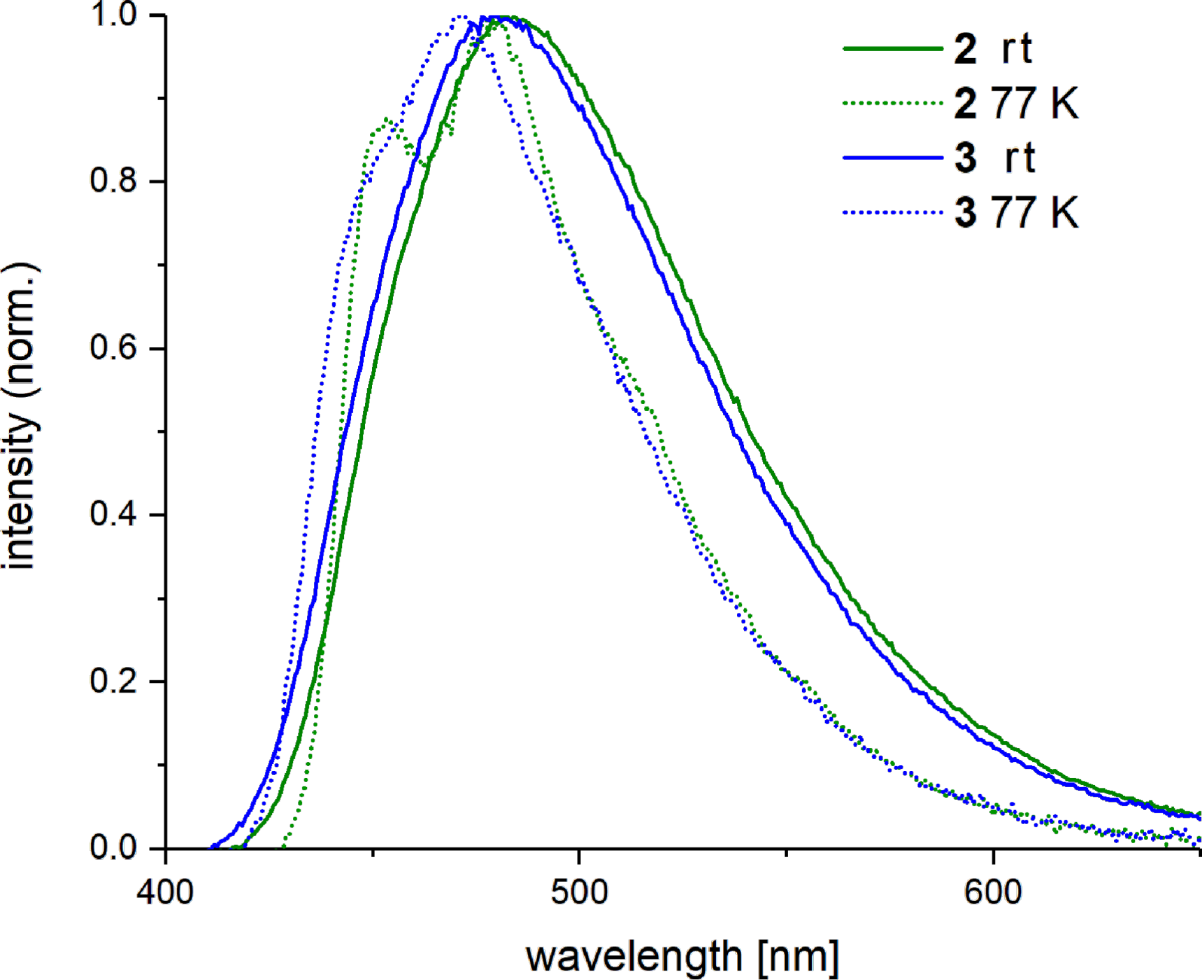
Emission spectra of complexes **2** and **3** measured at room temperature and 77 K, 2 wt % in a PMMA matrix and 0.5 mM in 2-MeTHF, respectively (λ_exc_ = 320 nm).

Photoluminescence spectra (Figure 3) were measured at ambient temperature in a PMMA matrix (2 wt % complex) and at 77 K in 2-MeTHF (0.5 mM). The room-temperature emission spectra of both complexes exhibit one broad, structurally unresolved band in the sky-blue spectral region.

The low-temperature emission maxima of both complexes display only a minor hypsochromic shift compared to the emission at ambient temperature: 5 nm for complex **2** and 8 nm for complex **3**. The emission profile of the mesityl complex **2** shows a vibronic progression with a spacing of 400 cm^−1^ between the first and second band. The low-temperature emission profile of duryl complex **3** mostly remains structurally unresolved. For both complexes, very high quantum yields of 82% (**2**) and 73% (**3**) at ambient temperatures as well as short decay times around 3 μs (Table 1) were measured. The complexes show no aggregation behavior at higher concentrations (10 wt % in PMMA and 100% amorphous film measurements, see Figures S1, S2 and Tables S2, S3 in Supporting Information File 1), which can be assigned to the steric demand of the aryl-substituted diketonate counter ligand.

**Table 1:** Photoluminescence data of complexes **2** and **3** (2 wt % in PMMA, λ_exc_ = 320 nm) and literature-known compound Pt(MPIM)(acac).

	CIE (x;y)^a^	λ_em_ [nm]^b^	Φ [%]^c^	τ_ν_^d^	τ_0_^e^	*k*_r_^f^	*k*_nr_^g^

Pt(MPIM)(acac) [42]	0.190; 0.190	441	7	**–**	**–**	**–**	**–**
Pt(MPIM)(mes) (**2**)	0.196; 0.326	482	82	2.6	3.1	320.5	70.4
Pt(MPIM)(dur) (**3**)	0.191; 0.303	479	73	2.4	3.3	306.6	113.4

^a^CIE coordinates, ^b^maximum emission wavelength, ^c^absolute quantum yield ± 5% ^d^decay lifetimes τ_ν_ (excited by laser pulses 360 nm, 20 kHz) in μs, ^e^τ_0_ = τ_ν_/Φ in μs, ^f^*k*_r_ = Φ/τ_ν_ in 10^3^ s^−1^, ^g^*k*_nr_ = (1 − Φ)/τ_ν_ in 10^3^ s^−1^.

**Figure 4:**
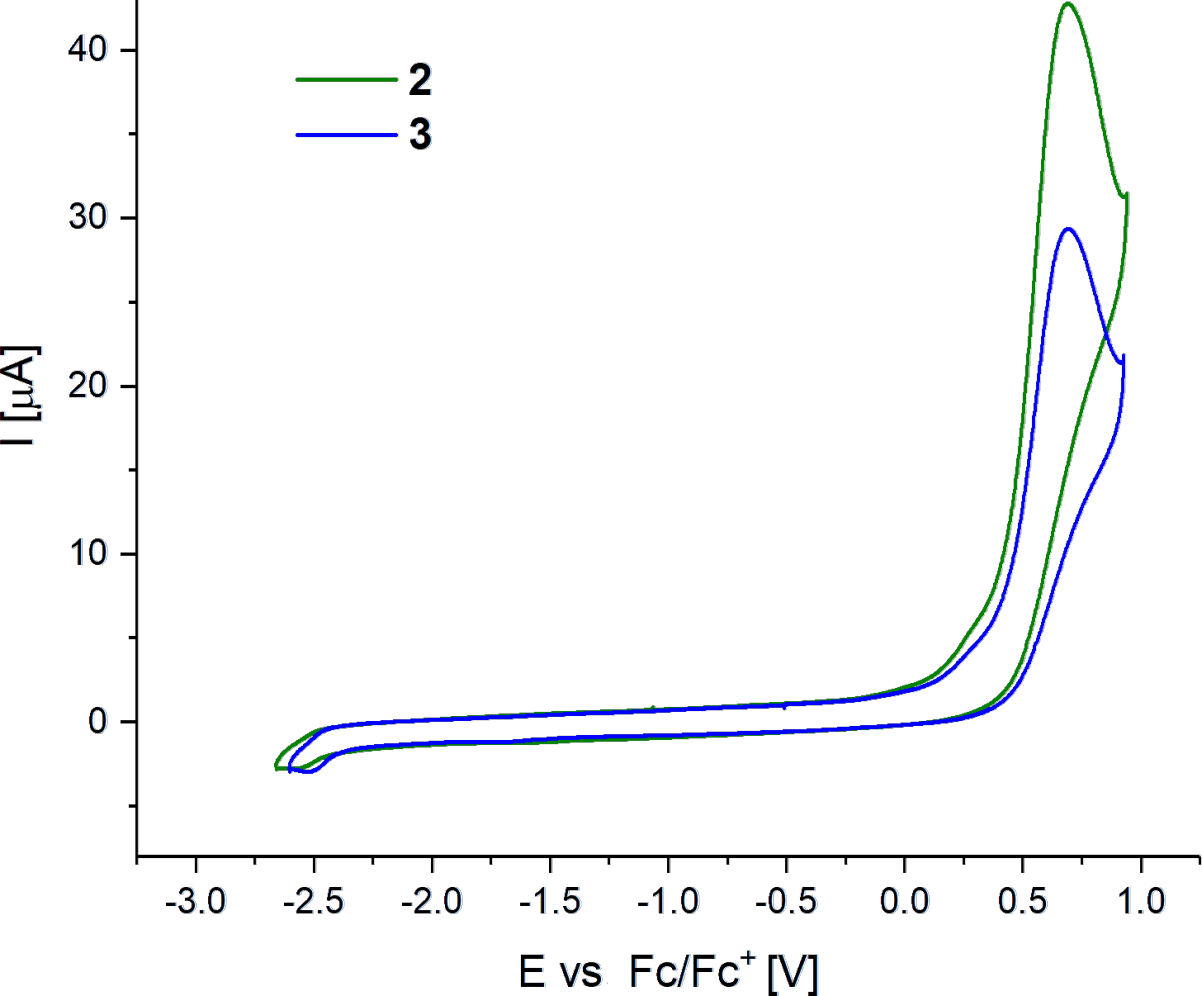
Cyclic voltammograms of complexes **2** and **3**, analyte concentration 10^−4^ M. Measured in DMF (0.1 M TBAP) vs Fc, *v* = 100 mV/s, under N_2_.

Cyclic voltammograms of complexes **2** and **3** were measured in DMF with ferrocene as an internal reference. For both compounds, one irreversible oxidation wave was measured (Figure 4), which is commonly found for platinum(II) complexes [16,44]. Irreversibility of the measured signals was confirmed by variation of the scan rate (30 mV/s to 1 V/s). The peak potential of the oxidation is located at 0.69 V vs ferrocene for both complexes. No reduction was observed for both complexes in the electrochemical window of the solvent. Thus, the electrochemical behavior of the newly synthesized substances is comparable.

## Discussion

**Figure 5:**
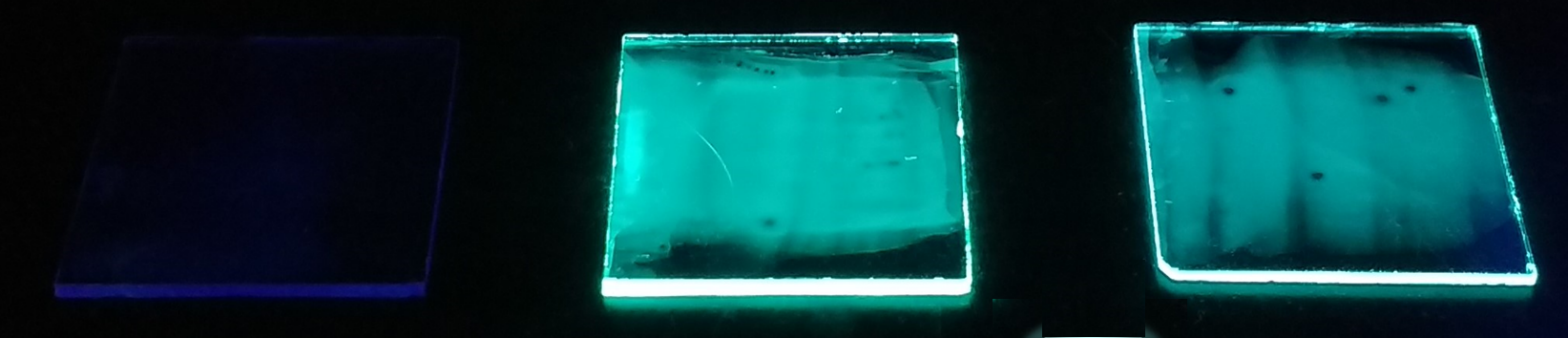
Thin films of Pt(MPIM)(acac) left, Pt(MPIM)(mes) (**2**) middle, and Pt(MPIM)(dur) (**3**) right, 2 wt % in PMMA under irradiation with ultraviolet light (365 nm).

Compared to the already published, structurally related 3-methyl-1-phenylimidazole platinum(II) complex with acetylacetonate as counter ligand, Pt(MPIM)(acac) [42], which shows a very weak emission (Φ = 7%), the new complexes exhibit a dramatically enhanced quantum yield (emission under UV irradiation is shown in Figure 5).

**Figure 6:**
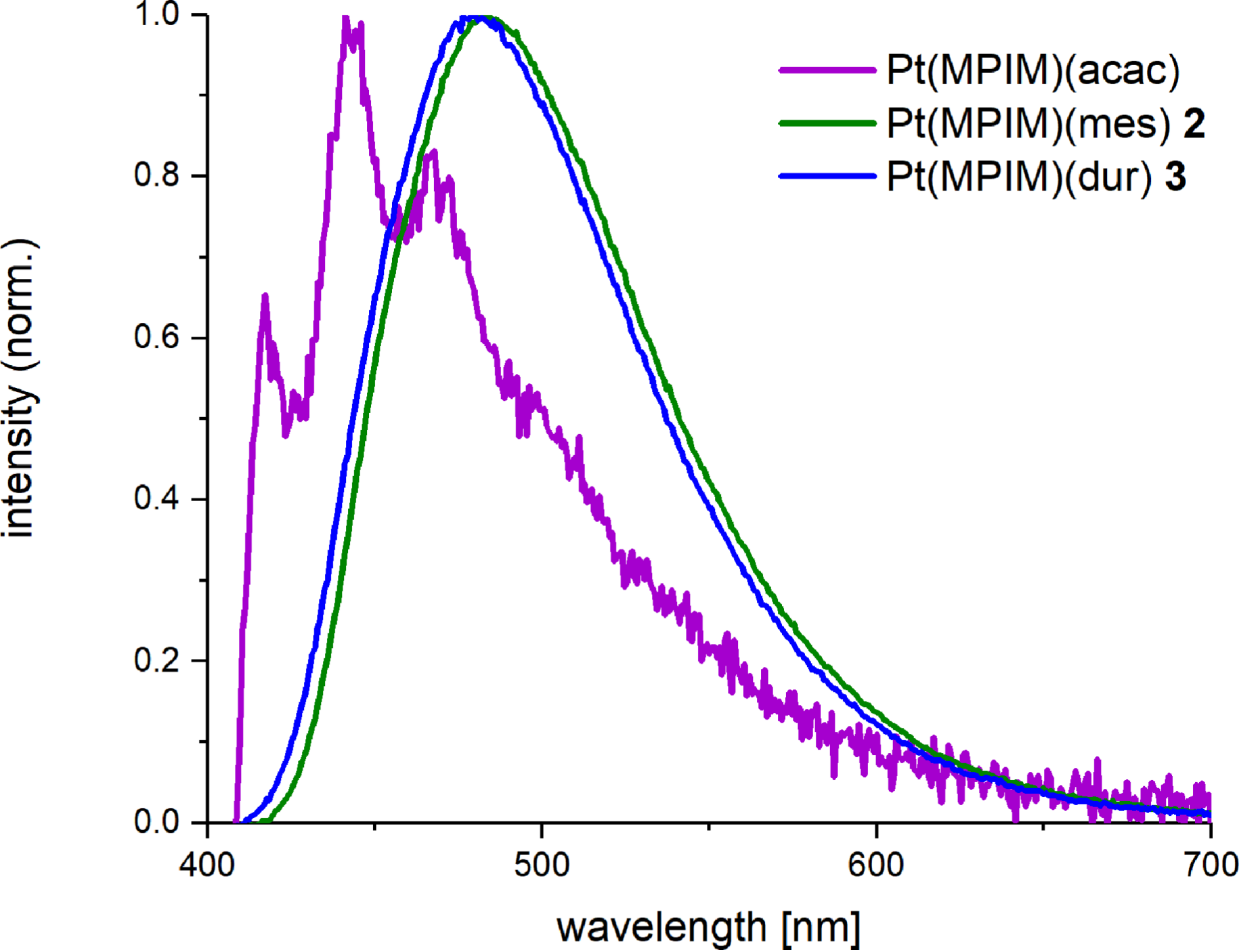
Photoluminescence spectra of **2** and **3** compared to the emission profile of Pt(MPIM)(acac), 2 wt % in PMMA, λ_exc_ = 320 nm.

The higher emission efficiency is accompanied by a red shift in emission color of about 40 nm (Figure 6). An improved quantum yield of Φ = 30% (5 wt % in PMMA) has already been reported for a 3-methyl-1-phenylimidazolium cyclometallated platinum(II) complex by the introduction of a sterically demanding ancillary ligand (α-duryl substituted acac) in the central position of the acetylacetonate between the two C=O groups [35]. Besides an increased quantum yield, the complex displayed a small red shift (λ_exc_ = 467 nm) compared to Pt(MPIM)(acac) and a decay time of 8.7 μs. When mesityl or duryl groups replace both methyl groups of the acetylacetonate, the quantum yield is further enhanced. Such a severe influence of the mesityl- and duryl-substituted auxiliary ligands on the quantum yield is unprecedented, although enhanced quantum yields have been reported for both ligands [18,39–41]. Additionally, the decay times of Pt(MPIM)(mes) and Pt(MPIM)(dur) are shorter compared to the phosphorescence decay of the α-duryl-substituted complex (8.7 µs at 77 K in 2-MeTHF).

**Figure 7:**
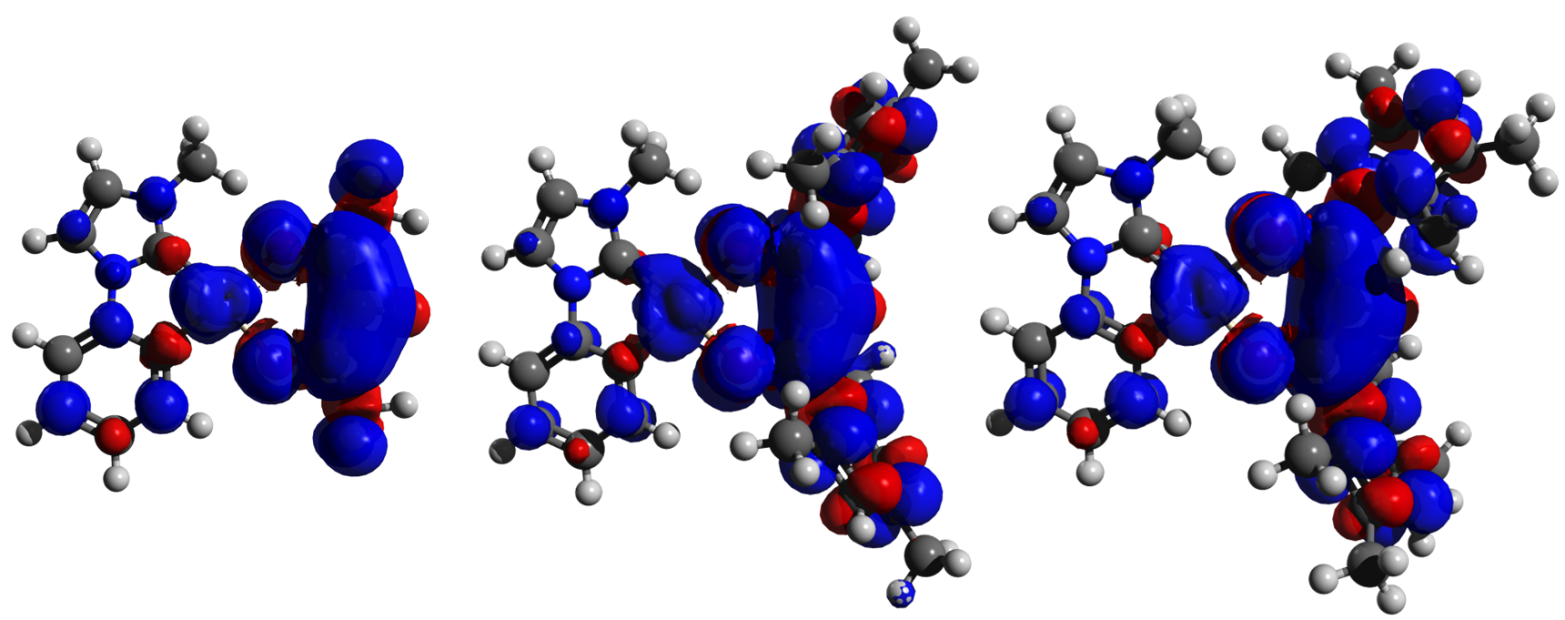
Localization of spin density on the complexes Pt(MPIM)(acac) left, Pt(MPIM)(mes) (**2**) middle, and Pt(MPIM)(dur) (**3**) right (B3LYP/6-31G(d), ECP LANL2DZ, isovalue 0.02).

The observed effects can be attributed to a major influence of the counter ligand on the emission characteristics, which is further supported by the localization of spin density almost exclusively on the ancillary ligand for all three complexes discussed. The spin densities were obtained from DFT calculations with the Gaussian 09 [45] program suite, using the B3LYP[46–50] functional and 6-31G(d) [51–56] basis set with Hay–Wadt ECP (LANL2DZ) [57–59] for platinum (Figure 7).

The observed red shift in emission color is also in agreement with the results of the DFT calculations (Supporting Information File 1, Table S4) of the predicted emission wavelength, according to a previously published procedure [60]. The bathochromic shift in emission color of complexes **2** and **3** can be assigned to the delocalization of electron density on the aryl-substituted auxiliary ligands.

## Conclusion

As shown above, we observed an unprecedented enhancement of the quantum yield for platinum(II) complexes with 3-methyl-1-phenylimidazole as C^C* cyclometalating ligand by changing the ancillary ligand from acetylacetonate (R = CH_3_) to sterically demanding aryl-substituted β-diketones (R = 2,4,6-trimethylphenyl, 2,3,5,6-tetramethylphenyl). The drastically increased quantum yield was accompanied by a shift in the emission color from the deep-blue to the sky-blue spectral region. Besides a very efficient phosphorescent emission, the two newly synthesized complexes also exhibit very short decay times of less than 3 μs. The profound impact of the counter ligand on the complexes’ emission properties originates from the diketonate ligand, which was also confirmed by DFT calculations.

## Experimental

Both complexes were characterized by ^1^H, ^13^C, and ^195^Pt NMR spectroscopy, ESIMS, and elemental analysis. Formation of the carbene complexes was verified by the disappearance of the characteristic NCHN proton signal of the imidazolium salt in the ^1^H NMR experiment. The syntheses of the platinum complexes were performed under an argon atmosphere and by exclusion of light, using flame-dried Schlenk tubes. Solvents of at least 99.0% purity were used. DMF was dried according to standard methods and stored over molecular sieve (3 Å) under argon atmosphere. Dichloro(1,5-cyclooctadiene)platinum(II) [Pt(COD)Cl_2_] was prepared following a modified literature procedure [61]. Potassium tetrachloroplatinate(II) was purchased from Pressure Chemicals (USA) and was used as received. Other chemicals were obtained from common suppliers and used without further purification. ^1^H, ^13^C, and ^195^Pt NMR spectra were recorded on a Bruker NMR spectrometer. ^1^H and ^13^C spectra were referenced internally using the resonances of the residual solvent (^1^H: 2.50 ppm, ^13^C: 39.52 ppm for DMSO-*d*_6_ and ^1^H: 7.26 ppm, ^13^C: 77.00 ppm for CDCl_3_). ^195^Pt spectra were referenced externally using potassium tetrachloroplatinate(II) in D_2_O (−1617.2 (PtCl_4_^2−^), −2654.1 ppm (PtCl_2_)). Chemical shifts are given in ppm downfield from TMS, coupling constants *J* in Hz (the signal splitting is abbreviated as followed: s = singlet, d = doublet, t = triplet, m = multiplet). Elemental analyses were performed by the analytical laboratory of the department using a Eurovektor Hekatech EA-3000 elemental analyzer. Melting points were measured on a Wagner and Munz Poly Therm A system and are not corrected.

### X-ray crystallography

Crystallographic data for compound **3** were collected on Bruker D8 VENTURE Kappa Duo PHOTON200 by IμS micro-focus sealed tube Mo Kα 0.71073 Å at a temperature of 100(2) K. The absorption corrections were carried out using numerical methods. The structure was solved by direct methods (XP) and refined by full matrix least squares based on *F*^2^ (SHELXL2014).

### Photophysical characterization

Absorption spectra of all complexes were measured on a Perkin Elmer lambda 25 spectrophotometer in dichloromethane solution. Photoluminescence measurements were performed in amorphous PMMA thin films doped with the emitter. Films were prepared by doctor blading a solution of 2 wt % emitter in a 10 wt % PMMA solution in dichloromethane on a quartz substrate with a 60 μm doctor blade. Film emission was measured under nitrogen flux. Excitation was carried out at different wavelengths (Xe-lamp with monochromator) and the emission was detected with a calibrated quantum-yield detection system (Hamamatsu, model C11347). The phosphorescence decay of all complexes was measured with an Edinburgh Instruments *mini-τ* by excitation with a pulsed EPLED (360 nm, 20 kHz) and time-resolved photon counting (TCSPC). Frozen 2-MeTHF glass emission samples at 77 K were prepared by inserting a sealed quartz tube, containing the solution under argon atmosphere, into liquid nitrogen. Spectroscopic grade 2-methyltetrahydrofuran (2-MeTHF) was purchased from ABCR and used as received.

### Cyclic voltammetry

Electrochemical measurements were performed with a BioLogic SP-150 potentiostat in degassed, dry *N,N*-dimethylformamide using a Pt counter electrode, a glassy carbon working electrode, and a Ag/Ag^+^ pseudo reference electrode. All complexes were measured as 0.1 mM solutions with the addition of 0.1 M (*n*-Bu)_4_NClO_4_ as supporting electrolyte at a sweep rate of 100 mV/s. Signals were identified as irreversible by varying the scan rate between 30 mV/s and 1 V/s. All measurements were internally referenced against the Fc/Fc^+^ redox couple. For visualization, the EC-Lab software V11.01 and Origin 2017 were used.

### Synthesis

#### (SP-4-4)-[1-Methyl-3-phenyl-1*H*-imidazolin-2-yliden-κ^C2^,κ^C2‘^][dimesitoylmethanato-κ^O^,κ^O’^]platinum(II) (**2**)

**General procedure:** A flame-dried and argon-flushed Schlenk tube was charged with 1-methyl-3-phenyl-1*H*-imidazol-3-ium iodide (**1**, 230 mg, 0.8 mmol) and silver(I) oxide (100 mg, 0.4 mmol). After the addition of 20 mL of dry DMF the reaction mixture was stirred under an argon atmosphere with the exclusion of light for two hours at room temperature, then for 23 hours at 50 °C. Dichloro(1,5-cyclooctadiene)platinum(II) (300 mg, 0.8 mmol) was added at room temperature, and the mixture was stirred for two hours at 50 °C, then for 24 hours at 120 °C. Afterwards, potassium *tert*-butanolate (180 mg, 1.6 mmol) and 1,3-bis(2,4,6-trimethylphenyl)propane-1,3-dione (495 mg, 1.4 mmol) were added, and the mixture was stirred for 24 hours at room temperature and then for six hours at 110 °C; all volatiles were removed in vacuo, the crude product was washed with water and purified by flash chromatography (silica gel, DCM/isohexanes 3:1). Afterwards, it was washed with pentane and cold ethanol. The residue was fully dissolved in ethanol and recrystallized. After washing again with cold ethanol and drying in vacuo*,* the pure product was obtained as yellow crystals in 5% yield (25 mg, 0.04 mmol). Mp. 134 °C; ^1^H NMR (CDCl_3_, 300 MHz) δ (ppm) 7.71 (dd, *pseudo*-t *J*_H,Pt_ = 24.9 Hz, *J* = 7.3 Hz, *J* = 1.2 Hz, 1H, CH_arom_), 7.26 (d, *J* = 2.1 Hz, 1H, CH_arom_), 7.06–6.88 (m, 3H, CH_arom_), 6.85 (d, *J* = 9.4 Hz, 4H, CH_arom_), 6.79 (d, *J* = 2.1 Hz, 1H, CH_arom_), 5.68 (s, 1H, CH), 3.94 (s, 3H, NCH_3_), 2.34 (d, *J* = 9.1 Hz, 12H, CH_3_), 2.30 (d, *J* = 4.3 Hz, 6H, CH_3_); ^13^C NMR (DMSO-*d*_6_, 75MHz) δ (ppm) 184.5 (CO), 183.6 (CO), 147.2 (NCN), 146.9 (C_arom_), 139.2 (C_arom_), 138.8 (C_arom_), 137.3 (C_arom_), 137.1(C_arom_), 133.2 (C_arom_), 132.9 (C_arom_), 130.7 (CH_arom_), 128.2 (CH_arom_), 128.1 (CH_arom_), 124.5 (C_arom_), 123.5 (CH_arom_), 123.5 (CH_arom_), 122.3 (CH_arom_), 115.4 (CH_arom_), 110.8 (CH_arom_), 106.5 (CH), 34.0 (NCH_3_), 20.7 (CH_3_), 20.6 (CH_3_), 19.4 (CH_3_), 19.2 (CH_3_); ^195^Pt NMR (DMSO-*d*_6_, 64.52 MHz,) δ (ppm) −3368. ESIMS *m*/*z* = 660.4 [M + H]^+^; anal. calcd for C_31_H_32_N_2_O_2_Pt: C, 56.44; H, 4.89; N, 4.25; found: C, 56.68; H, 5.08; N, 4.16.

#### (SP-4-4)-[1-Methyl-3-phenyl-1*H*-imidazolin-2-yliden-κ^C2^,κ^C2‘^][bis(2,3,5,6-tetramethylphenyl) propane-1,3-dionato-κ^O^,κ^O‘^]platinum(II) (**3**)

The product was obtained following the general procedure reported for **2** using 1-methyl-3-phenyl-1*H*-imidazol-3-ium iodide (**1**, 230 mg, 0.8 mmol) and silver(I) oxide (100 mg, 0.4 mmol), dichloro(1,5-cyclooctadiene)platinum(II) (300 mg, 0.8 mmol) together with potassium *tert*-butanolate (180 mg, 1.6 mmol) and the β-diketonate 1,3-bis(2,3,5,6-tetramethylphenyl)propane-1,3-dione (540 mg, 1.6 mmol). The crude product was washed with water, isolated by flash chromatography (silica gel, DCM/isohexanes 4:1), and washed with pentane and cold ethanol. The residue was completely dissolved in ethanol and recrystallized. After washing with cold ethanol again and drying in vacuo at 50 °C, the pure product was obtained as a yellow powder in 18% yield (79 mg, 0.14 mmol). Mp. decomp. >310 °C; ^1^H NMR (CDCl_3_, 300 MHz) δ (ppm) 7.72 (dd, *pseudo*-t *J*_H,Pt_ = 26.4 Hz, *J* = 7.4 Hz, *J* = 0.9 Hz, 1H, CH_arom_), 7.26 (d, *J* = 2.1 Hz, 1H, CH_arom_), 7.06–6.88 (m, 5H, CH_arom_), 6.79 (d, *J* = 2.1 Hz, 1H, CH_arom_), 5.64 (s, 1H, CH), 3.93 (s, 3H, NCH_3_), 2.31–2.16 (m, 24H, CH_3_); ^13^C NMR (CDCl_3_, 75 MHz) δ (ppm) 185.8 (CO), 185.6 (CO), 146.8 (NCN), 142.4 (C_arom_), 134.1 (C_arom_), 133.7 (C_arom_), 133.6 (C_arom_), 132.2 (CH_arom_), 131.1 (CH_arom_), 131.0 (CH_arom_), 129.9 (C_arom_), 129.8 (C_arom_), 129.5 (C_arom_), 124.3 (C_arom_), 124.1 (CH_arom_), 123.6 (CH_arom_), 120.8 (CH_arom_), 114.3 (CH_arom_), 109.9 (CH_arom_), 107.5 (CH), 35.0 (CH_3_), 19.7 (CH_3_), 19.7 (CH_3_), 16.5 (CH_3_), 16.3 (CH_3_); ^195^Pt NMR (CDCl_3_, 64.52 MHz) δ (ppm) −3383; ESIMS *m*/*z* = 688.4 [M + H]^+^; anal. calcd for C_33_H_36_N_2_O_2_Pt: C, 57.63; H, 5.28; N, 4.07; found: C, 57.93; H, 5.46; N, 3.82.

## Supporting Information

The Supporting Information contains NMR-spectra, additional figures, details of the solid state structure determination and computational details. CCDC 1823322 contains the supplementary crystallographic data for this paper. These data can be obtained free of charge via http://www.ccdc.cam.ac.uk/data_request/cif, or by emailing data_request@ccdc.cam.ac.uk, or by contacting The Cambridge Crystallographic Data Centre, 12 Union Road, Cambridge CB21EZ, UK; fax: +44 1223 336033.

File 1 NMR spectra, additional figures, details of the solid state structure determination and computational details.

File 2 Crystallographic data.
