# Supplementary material for: Enhanced quantum yields by sterically demanding aryl-substituted β-diketonate ancillary ligands
Source: Beilstein J Org Chem. 2018 Mar 21;14:664–71. doi: 10.3762/bjoc.14.54 (PMC5870149; doi:10.3762/bjoc.14.54)
Supplement: File 1 — NMR spectra, additional figures, details of the solid state structure determination and computational details. [file Beilstein_J_Org_Chem-14-664-s001.pdf]

Supporting Information

for

Enhanced quantum yields by sterically demanding aryl-substituted β-diketonate ancillary ligands

Rebecca Pittkowski and Thomas Strassner*

Address: Physical Organic Chemistry, Technische Universität Dresden, Bergstraße, 01069 Dresden, Germany.

Email: Thomas Strassner - thomas.strassner@chemie.tu-dresden.de

*Corresponding author

NMR spectra, additional figures, details of the solid state structure determination and computational details

Details of the solid-state structure determination S2

Photophysical properties S3

NMR spectra S5

Computational details S7

Frontier molecular orbital S8

XYZ-coordinates S9

References S14

Details of the solid-state structure determination

Table S1: Crystal data and crystallographic details for complex **3**

complex	**3**
empirical formula	$C_{33}H_{36}N_2O_2Pt$
formula weight [g/mol]	687.73
T [K]	100(2)
wavelength [Å]	0.71073
crystal system	Monoclinic
space group	$P\ 2_1/n$
a [Å]	9.8385 (19)
b [Å]	27.837 (6)
c [Å]	11.285 (2)
α [°]	90.0
β [°]	110.846 (7)
γ [°]	90.0
V [Å^3]	2888.4 (10)
Z	4
D_{calc} [Mg/m^3]	1.582
μ(MoKα) [mm^{-1}]	4.889
crystal size [mm^3]	0.91 × 0.87 × 0.52
F(000)	1368
reflections collected	209519
independent reflections	6641
Goodness-of-fit on F^2	1.163
R_1 [I>2σ(I)]	0.0271
wR_2 [I>2σ(I)]	0.0592
data / restraints / parameters	6641 / 0 / 352
$\Delta\rho_{max}$, $\Delta\rho_{min}$ (e Å^{-3})	27.53, 2.33

Photophysical properties

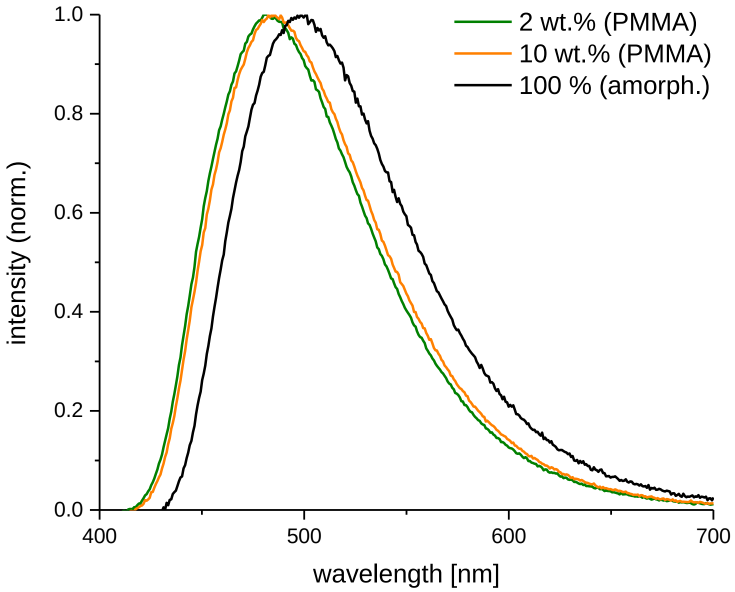

Figure S1: Emission profile of complex **3** measured in different concentrations (λ_{exc} = 320 nm).

Table S2: Photoluminescence data of complex **2** (λ_{exc} = 320 nm)

Complex conc.	**CIE (x;y)**[a]	**λ_{em} [nm]**[b]	**Φ [%]**[c]	**τ [µs]**[d]
2 wt.% (in PMMA)	0.196; 0.326	482	82	3.1
10 wt.% (in PMMA)	0.203; 0.345	485	77	2.4
100 wt.% (amorphous)	0.238; 0.429	500	36	3.5

[a] CIE coordinates, [b] maximum emission wavelength, [c] absolute quantum yield ± 5% [d] decay lifetimes (excited by laser pulses 360 nm, 20 kHz) given as $\tau = \tau_{exp} / \Phi$.

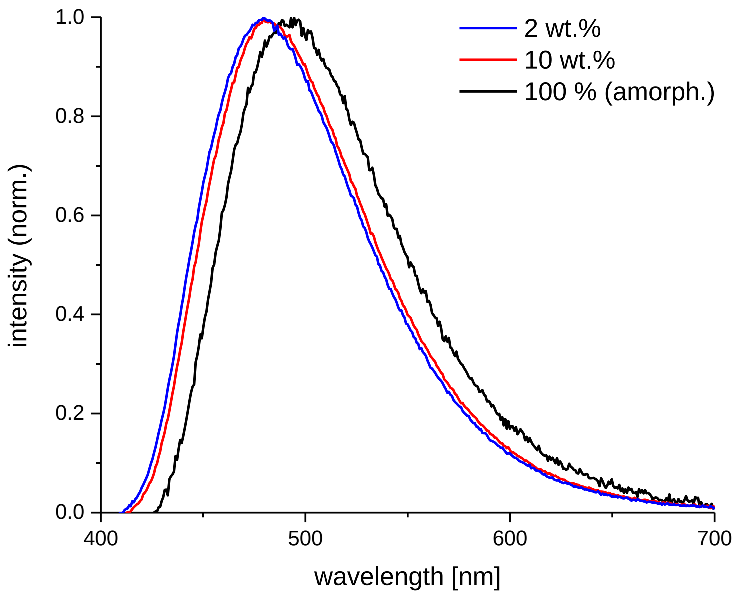

Figure S2: Emission profile of complex **3** measured in different concentrations (λ_{exc} = 320 nm).

Table S3: Photoluminescence data of complex **3** in different concentrations (λ_{exc} = 320 nm)

Complex Conc.	**CIE (x;y)**[a]	**λ_{em} [nm]**[b]	**Φ [%]**[c]	**τ [μs]**[d]
2 wt.% (in PMMA)	0.191; 0.303	479	73	3.3
10 wt.% (in PMMA)	0.196; 0.321	479	72	2.6
100 wt.% (amorphous)	0.221; 0.384	494	26	5.7

[a] CIE coordinates, [b] maximum emission wavelength, [c] absolute quantum yield ± 5% [d] decay lifetimes (excited by laser pulses 360 nm, 20 kHz) given as $\tau = \tau exp / \Phi$.

NMR spectra

2 – Pt(MPIM)(mes)

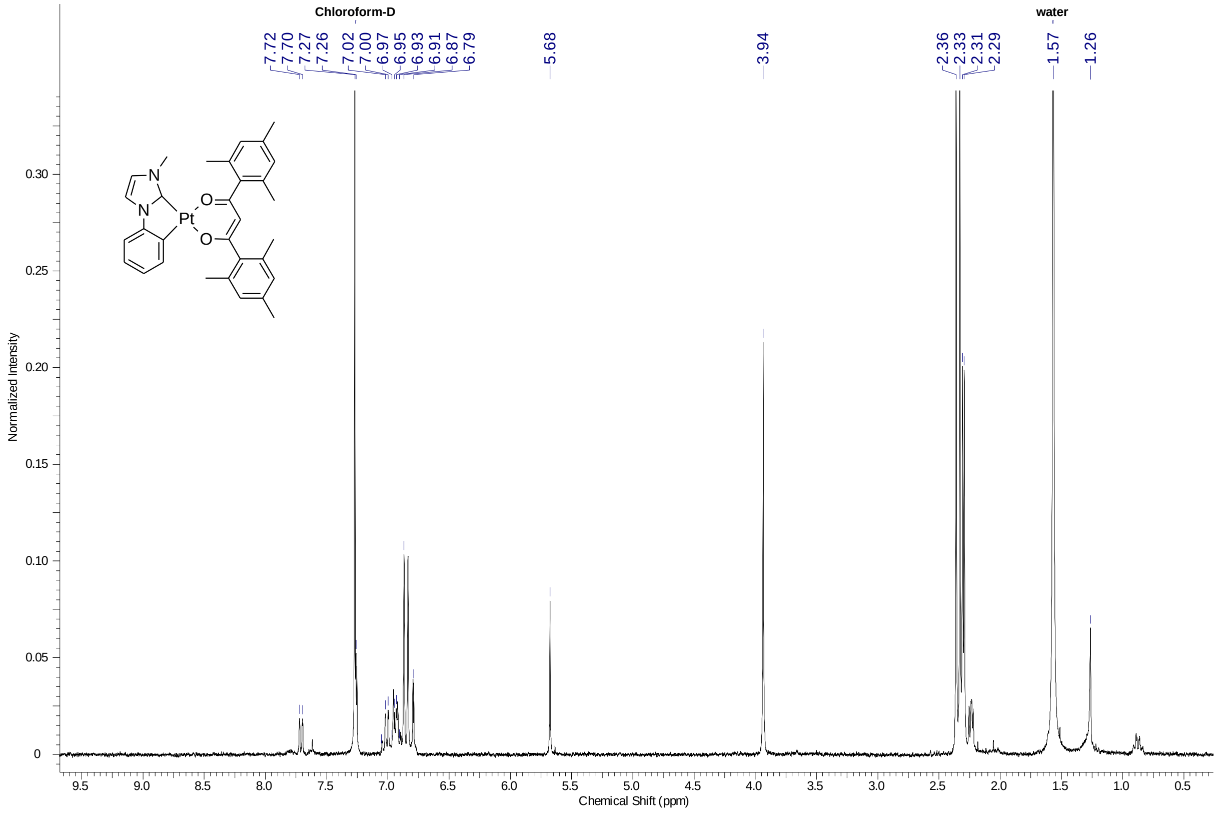

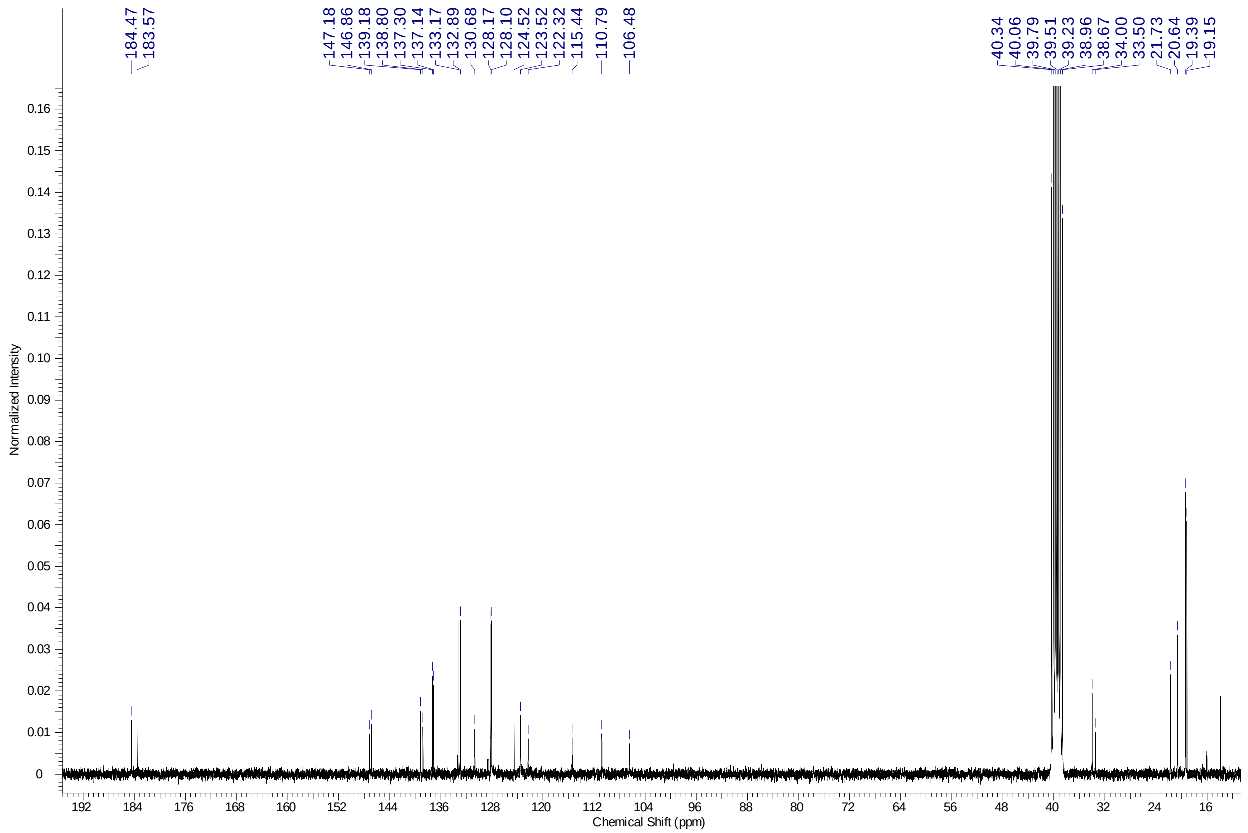

3 – Pt(MPIM)(dur)

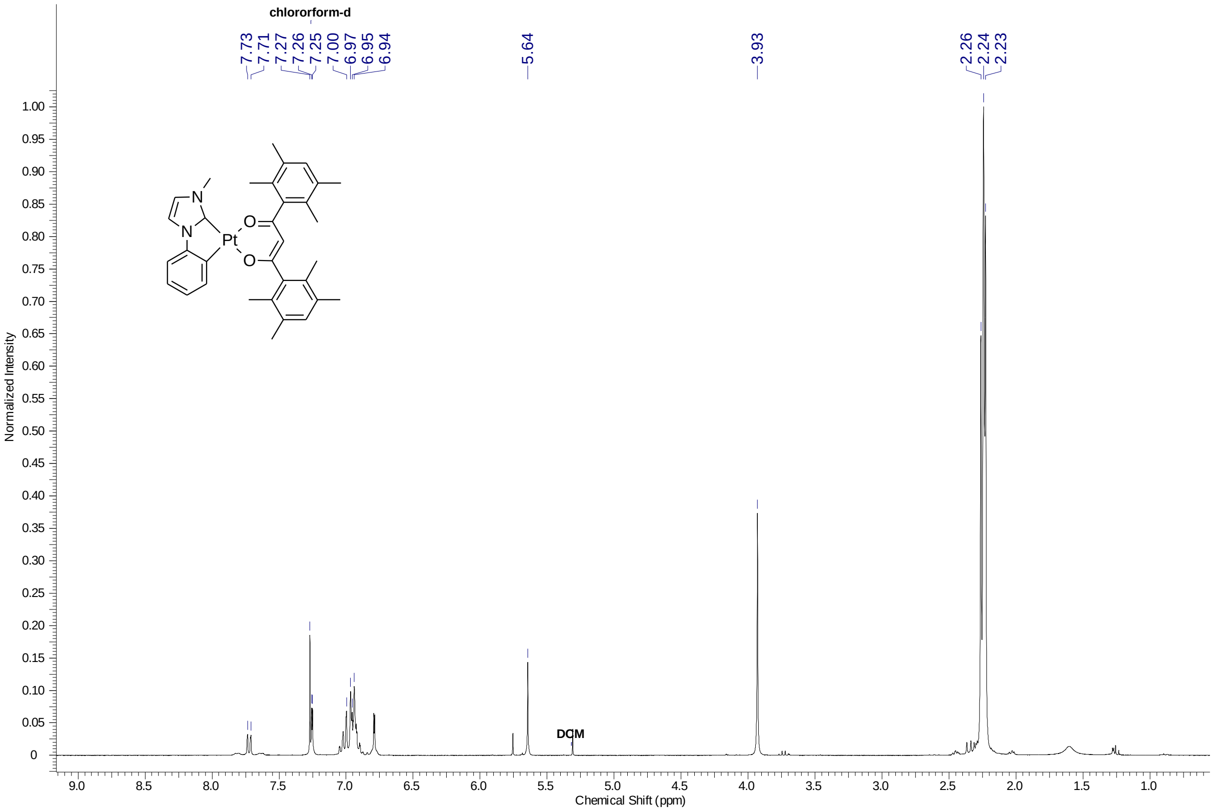
chlororform-d
7.73
7.71
7.27
7.26
7.25
7.00
6.97
6.95
6.94
5.64
3.93
2.26
2.24
2.23
DCM
Normalized Intensity
Chemical Shift (ppm)
N
N
Pt
O
O

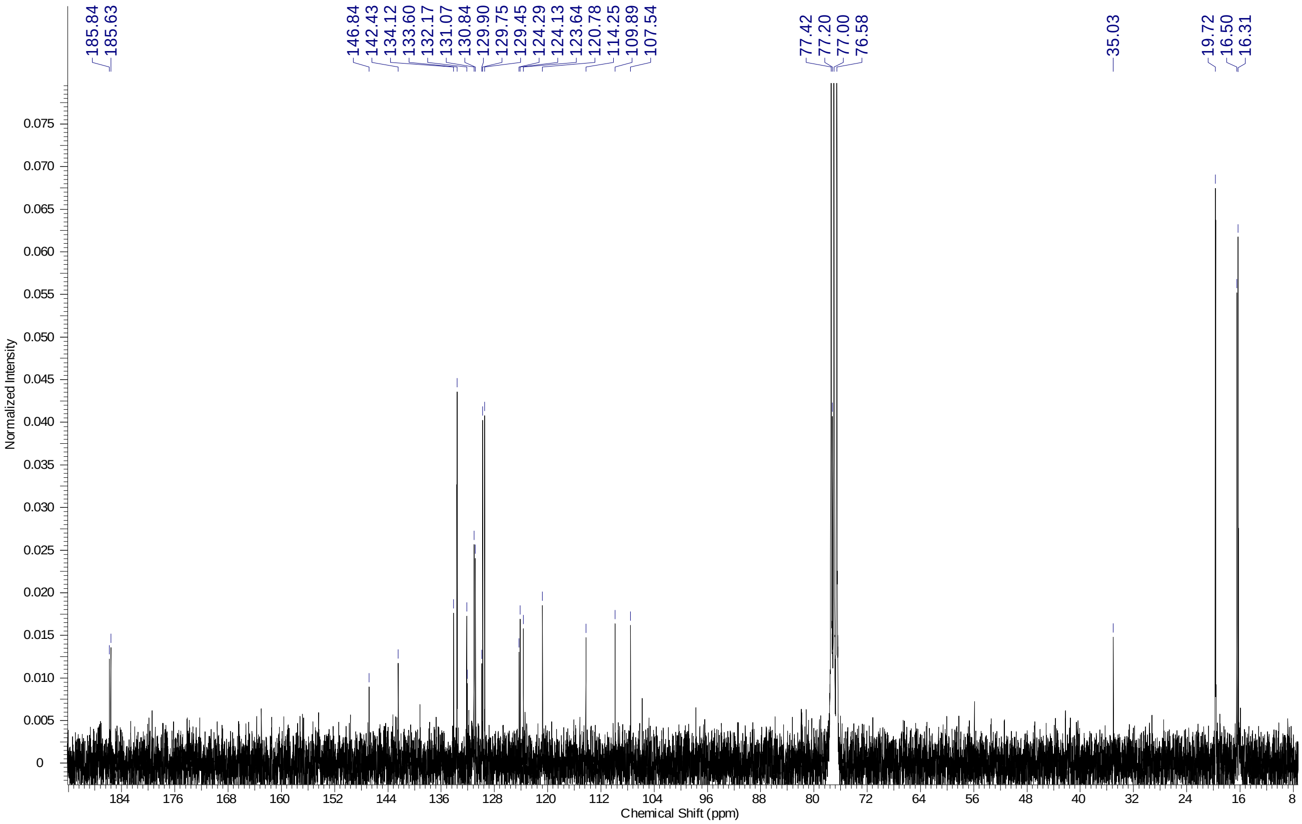
185.84
185.63
146.84
142.43
134.12
133.60
132.17
131.07
130.84
129.90
129.75
129.45
124.29
124.13
123.64
120.78
114.25
109.89
107.54
77.42
77.20
77.00
76.58
35.03
19.72
16.50
16.31
Normalized Intensity
Chemical Shift (ppm)

Computational details

All calculations were performed with Gaussian 09 [1] using the B3LYP [2-6] functional with the 6-31G(d) [7-12] basis set and Hay-Wadt ECP (LANL2DZ)[13-15]. All structures were verified as true minimum geometries by the absence of negative eigenvalues in the vibrational frequency analysis. Frontier molecular orbitals (FMO) were obtained from the calculated singlet geometries. The calculation of spin densities was performed based on the obtained triplet geometries. The calculation of the theoretical emission wavelengths was performed using a previously published method [16]. GaussView [17], CYLview [18], and Avogadro [19] were used for visualization.

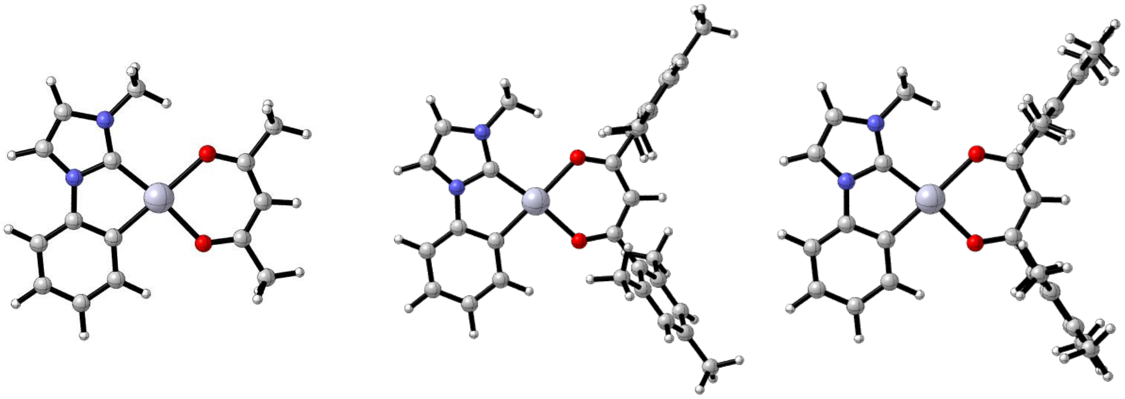

Figure S3: Optimized singlet geometries of Pt(MPIM)(acac) left, Pt(MPIM)(mes) middle, and Pt(MPIM)(dur) right.

Table S4: Comparison of calculated and experimentally obtained emission maxima for complexes **2** and **3** as well as Pt(MPIM)(acac).

**Complex**	**λ_{em} (theo)[a]**	**λ_{em} (exp)[b]**
Pt(MPIM)(acac) [2]	432	441
Pt(MPIM)(mes) **2**	458	482
Pt(MPIM)(dur) **3**	451	479

[a] calculated according to ref.[2] (B3LYP/6-31G(d), ECP LANL2DZ) [b] 2 wt% in PMMA, RT, N2, λ_{exc} = 320 nm.

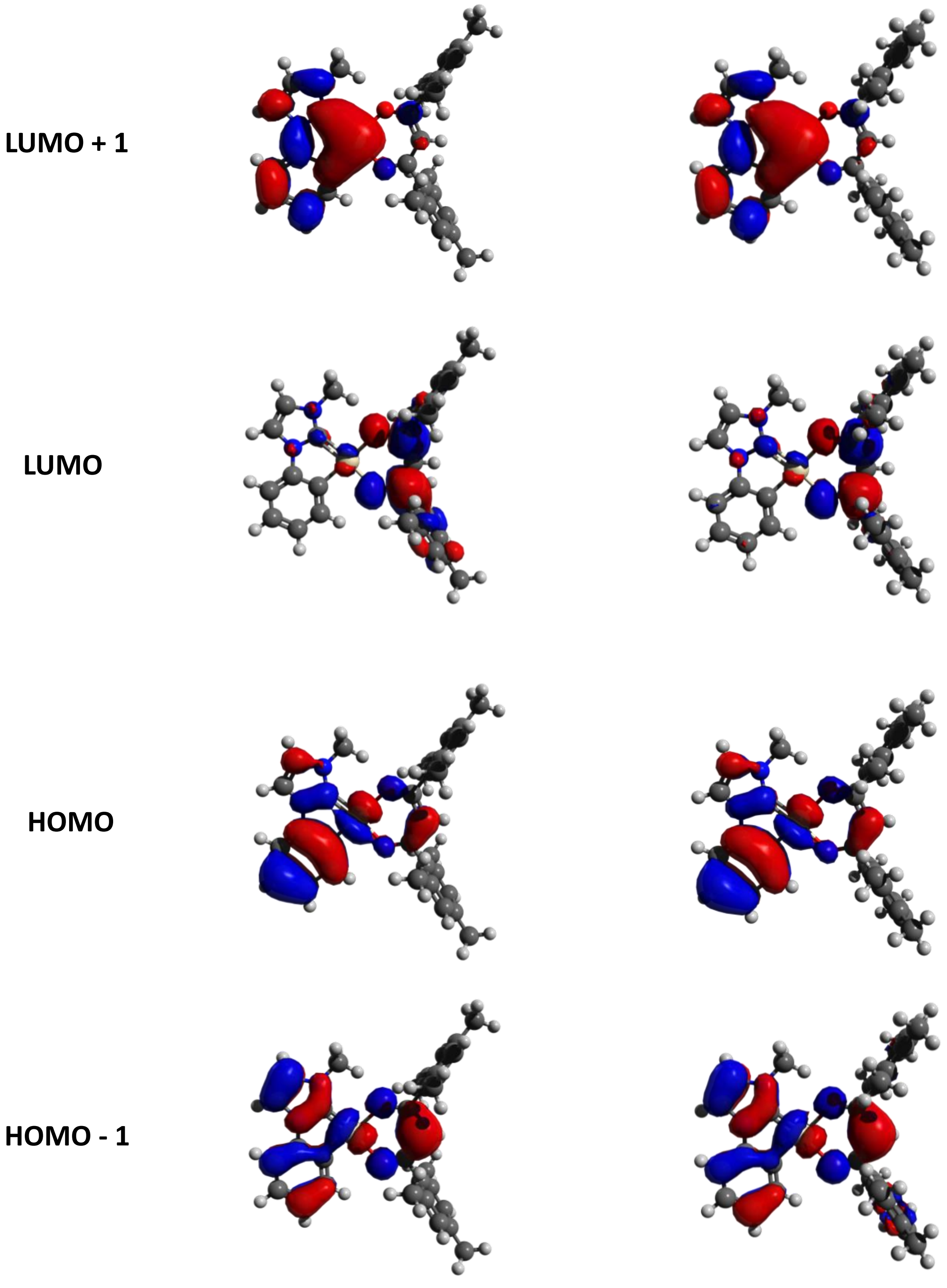

Figure S4: Representation of the frontier molecular orbitals *(FMOs)* of **2** (left) and **3** (right), obtained from the calculated singlet geometries (B3LYP/6-31G(d), ECP LANL2DZ, Isovalue 0.02).

XYZ-coordinates

XYZ-coordinates for the singlet ground states of the computed compounds (B3LYP/6-31G(d), ECP LANL2DZ)

Pt(MPIM)(acac)

Pt	-0.25930	-0.04001	0.00002
C	1.39469	-1.17210	-0.00028
C	1.47336	-2.56694	-0.00048
C	2.61364	-0.46501	-0.00024
C	2.71662	-3.21358	-0.00061
H	0.55585	-3.14744	-0.00052
C	3.85954	-1.08101	-0.00030
C	3.90372	-2.47898	-0.00051
H	2.75703	-4.30023	-0.00079
H	4.77885	-0.50062	-0.00013
H	4.86438	-2.98637	-0.00056
C	1.11385	1.36377	0.00012
C	3.28202	2.02136	0.00032
C	2.50489	3.13699	0.00039
H	4.35444	1.91108	0.00012
H	2.77218	4.18224	0.00055
O	-2.01640	1.21716	0.00025
C	-3.21959	0.79024	-0.00059
O	-1.52545	-1.70609	0.00006
C	-2.80254	-1.68749	0.00036
N	1.17625	2.71906	0.00039
N	2.41547	0.94413	0.00006
C	0.02811	3.61990	0.00087
H	0.05400	4.25287	0.89371
H	0.05470	4.25460	-0.89074
H	-0.88241	3.02035	-0.00007
C	-4.27582	1.87954	-0.00185
H	-5.29321	1.48060	-0.00306
H	-4.14446	2.51669	0.88058
H	-4.14229	2.51660	-0.88401
C	-3.63221	-0.55285	-0.00028
H	-4.70077	-0.73345	-0.00050

C	-3.43956	-3.06305	0.00174
H	-3.10261	-3.61867	0.88437
H	-4.53153	-3.02171	0.00062
H	-3.10076	-3.62143	-0.87841

Pt(MPIM)(mes) (**2**)

Pt	-1.35900	-0.32519	0.03017
C	-3.10057	0.66714	0.07154
C	-3.29361	2.05013	0.11504
C	-4.25639	-0.13884	0.05803
C	-4.58631	2.59097	0.14103
H	-2.42782	2.70527	0.13111
C	-5.54879	0.37174	0.08292
C	-5.70839	1.76071	0.12449
H	-4.71614	3.66999	0.17502
H	-6.41686	-0.28261	0.07121
H	-6.70773	2.18635	0.14463
C	-2.61163	-1.83847	0.00240
C	-4.71809	-2.67104	-0.01170
C	-3.85185	-3.71848	-0.04707
H	-5.79594	-2.64948	-0.00573
H	-4.03212	-4.78175	-0.07742
O	0.48798	-1.43955	-0.00891
C	1.65420	-0.91572	-0.01410
O	-0.23541	1.44088	0.04795
C	1.04059	1.52497	0.03255
N	-3.94283	-1.52676	0.01836
C	-1.34444	-3.99904	-0.07009
H	-1.31086	-4.65443	0.80599
H	-1.33004	-4.61107	-0.97747
H	-0.48328	-3.33083	-0.06316
C	1.95649	0.45679	0.00544
H	3.00797	0.72173	-0.01305
C	1.58157	2.93145	0.01452
C	2.15477	3.49128	1.17182
C	1.46997	3.69089	-1.16807
C	2.62253	4.80969	1.12029
C	1.96061	4.99939	-1.17615

C	2.54337	5.57790	-0.04377
C	3.08858	6.98692	-0.08643
C	2.79814	-1.89947	-0.04566
C	3.29548	-2.42600	1.16193
C	3.32690	-2.31838	-1.28072
C	4.33375	-3.36142	1.11124
C	4.36425	-3.25747	-1.28482
C	4.88604	-3.78712	-0.10113
C	6.02991	-4.77485	-0.12818
H	4.77208	-3.58458	-2.23955
C	2.78623	-1.77642	-2.58594
H	1.70461	-1.93477	-2.66714
H	2.95534	-0.69633	-2.67387
H	3.26623	-2.26273	-3.44081
C	2.72023	-1.99770	2.49423
H	2.85338	-0.92198	2.66224
H	1.64301	-2.19333	2.54620
H	3.20351	-2.52977	3.31944
H	4.71832	-3.76976	2.04420
C	2.25420	2.71203	2.46615
H	1.29985	2.24272	2.73003
H	2.99862	1.90900	2.40227
H	2.54594	3.36963	3.29111
H	3.05506	5.24759	2.01800
H	1.88040	5.58380	-2.09101
C	0.83565	3.11158	-2.41350
H	1.31390	2.16842	-2.70528
H	-0.22531	2.89097	-2.25427
H	0.92010	3.80688	-3.25430
H	6.05828	-5.33018	-1.07183
H	5.95418	-5.50015	0.68964
H	6.99782	-4.26669	-0.02062
H	3.11259	7.43783	0.91138
H	2.48490	7.63145	-0.73508
H	4.11500	7.00532	-0.47743

Pt(MPIM)(dur) (**3**)

Pt	-1.49746	-0.37813	-0.00011
C	-3.25288	0.59023	-0.00014
C	-3.46558	1.97100	-0.00024
C	-4.39700	-0.23241	-0.00002
C	-4.76602	2.49358	-0.00023
H	-2.60889	2.63823	-0.00033
C	-5.69666	0.26001	-0.00001
C	-5.87615	1.64717	-0.00011
H	-4.91142	3.57117	-0.00031
H	-6.55530	-0.40679	0.00009
H	-6.88156	2.05882	-0.00010
C	-2.72832	-1.90960	0.00004
C	-4.82281	-2.77185	0.00020
C	-3.94181	-3.80753	0.00024
H	-5.90087	-2.76534	0.00026
H	-4.10705	-4.87368	0.00031
O	0.36398	-1.46948	-0.00002
C	1.52291	-0.93138	0.00003
O	-0.39821	1.40360	-0.00024
C	0.87609	1.50069	-0.00016
N	-2.65945	-3.26428	0.00014
N	-4.06379	-1.61638	0.00007
C	-1.43075	-4.05339	0.00013
H	-1.39798	-4.68666	0.89235
H	-1.39818	-4.68691	-0.89192
H	-0.57891	-3.37331	-0.00006
C	1.80670	0.44531	-0.00005
H	2.85391	0.72541	-0.00002
C	1.40462	2.91593	-0.00014
C	1.63085	3.56219	1.23073
C	1.63196	3.56185	-1.23098
C	2.10952	4.88623	1.22433
C	2.11057	4.88591	-1.22452
C	2.34006	5.51650	-0.00008
H	2.70866	6.54095	-0.00005
C	2.68325	-1.89948	0.00016
C	3.20047	-2.34920	-1.23041
C	3.20090	-2.34844	1.23082

C	4.27562	-3.25866	-1.22412
C	4.27603	-3.25793	1.22470
C	4.78918	-3.69084	0.00034
H	5.62108	-4.39311	0.00040
C	4.87403	-3.76658	2.51608
H	5.25304	-2.94797	3.14179
H	4.13658	-4.30832	3.12319
H	5.70749	-4.44839	2.32022
C	2.63317	-1.87465	2.55170
H	1.75721	-1.23643	2.42321
H	2.33461	-2.72291	3.18069
H	3.37786	-1.30653	3.12537
C	2.63230	-1.87615	-2.55136
H	1.75621	-1.23808	-2.42294
H	3.37670	-1.30813	-3.12549
H	2.33377	-2.72476	-3.17990
C	4.87323	-3.76804	-2.51539
H	4.13560	-4.31010	-3.12199
H	5.25207	-2.94978	-3.14166
H	5.70674	-4.44975	-2.31939
C	1.36192	2.87341	2.55130
H	1.05532	1.83379	2.42328
H	2.25163	2.88086	3.19417
H	0.56593	3.38457	3.10869
C	2.37005	5.62769	2.51517
H	1.46657	5.70054	3.13470
H	3.13077	5.12704	3.12875
H	2.72073	6.64551	2.31782
C	2.37213	5.62707	-2.51533
H	3.13354	5.12643	-3.12806
H	1.46921	5.69954	-3.13571
H	2.72240	6.64503	-2.31794
C	1.36414	2.87272	-2.55160
H	1.05823	1.83290	-2.42354
H	0.56807	3.38324	-3.10945
H	2.25416	2.88069	-3.19403

References SI

[1] Frisch, M. J.; Trucks, G. W.; Schlegel, H. B.; Scuseria, G. E.; Robb, M. A.; Cheeseman, J. R.; Scalmani, G.; Barone, V.; Mennucci, B.; Petersson, G. A.; Nakatsuji, H.; Caricato, M.; Li, X.; Hratchian, H. P.; Izmaylov, A. F.; Bloino, J.; Zheng, G.; Sonnenberg, J. L.; Hada, M.; Ehara, M.; Toyota, K.; Fukuda, R.; Hasegawa, J.; Ishida, M.; Nakajima, T.; Honda, Y.; Kitao, O.; Nakai, H.; Vreven, T.; Montgomery Jr., J. A.; Peralta, J. E.; Ogliaro, F.; Bearpark, M.; Heyd, J. J.; Brothers, E.; Kudin, K. N.; Staroverov, V. N.; Kobayashi, R.; Normand, J.; Raghavachari, K.; Rendell, A.; Burant, J. C.; Iyengar, S. S.; Tomasi, J.; Cossi, M.; Rega, N.; Millam, J. M.; Klene, M.; Knox, J. E.; Cross, J. B.; Bakken, V.; Adamo, C.; Jaramillo, J.; Gomperts, R.; Stratmann, R. E.; Yazyev, O.; Austin, A. J.; Cammi, R.; Pomelli, C.; Ochterski, J. W.; Martin, R. L.; Morokuma, K.; Zakrzewski, V. G.; Voth, G. A.; Salvador, P.; Dannenberg, J. J.; Dapprich, S.; Daniels, A. D.; Farkas, Ö.; Foresman, J. B.; Ortiz, J. V.; Cioslowski, J.; Fox, D. J. *Gaussian 09*, Revision D.01; Gaussian Inc.: Wallingford CT, 2009.

[2] Vosko, S. H.; Wilk, L.; Nusair, M. Can. J. Phys. 1980, 58, 1200–1211.

[3] Becke, A. D. J. Chem. Phys. 1993, 98, 5648-5652.

[4] Miehlich, B.; Savin, A.; Stoll, H.; Preuss, H. Chem. Phys. Lett. 1989, 157, 200–206.

[5] Lee, C.; Yang, W.; Parr, R. G. Phys. Rev. B 1988, 37, 785–789.

[6] Stephens, P. J.; Devlin, F. J.; Chabalowski, C. F.; Frisch, M. J. J. Phys. Chem. 1994, 98, 11623–11627.

[7] Ditchfield, R.; Hehre, W. J.; Pople, J. A. J. Chem. Phys. 1971, 54, 724–728.

[8] Hehre, W. J.; Ditchfield, R.; Pople, J. A. J. Chem. Phys. 1972, 56, 2257–2261.

[9] Hariharan, P. C.; Pople, J. A. Chem. Phys. Lett. 1972, 16, 217–219.

[10] Hariharan, P. C.; Pople, J. A. Theor. Chim. Acta 1973, 28, 213–222.

[11] Hariharan, P. C.; Pople, J. A. Mol. Phys. 1974, 27, 209–214.

[12] Rassolov, V. A.; Pople, J. A.; Ratner, M. A.; Windus, T. L. J. Chem. Phys. 1998, 109, 1223–1229.

[13] Hay, P. J.; Wadt, W. R. J. Chem. Phys. 1985, 82, 270–283.

[14] Wadt, W. R.; Hay, P. J. J. Chem. Phys. 1985, 82, 284–298.

[15] Hay, P. J.; Wadt, W. R. J. Chem. Phys. 1985, 82, 299–310.

[16] Unger, Y.; Strassner, T.; Lennartz, C. J. Organomet. Chem. 2013, 748, 63–67.

[17] Dennington, R.; Keith, T. A.; Millam, J. M. *Gaussview*; Semichem Inc.: Shawnee Mission, KS, 2009.

[18] Legault, C. Y. *CYLview*, 1.0b; Université de Sherbrooke, 2009.

[19] Hanwell, M. D.; Curtis, D. E.; Lonie, D. C.; Vandermeersch, T.; Zurek, E.; Hutchison, G. R. J. Cheminform. 2012, 4, 1–17.
